# The interaction between candidate SNPs and social factors significantly influences the prevalence of alcohol use disorder in Chinese young male adults

**DOI:** 10.1371/journal.pone.0330822

**Published:** 2025-08-26

**Authors:** Niu Ju, Yilu Zhao, Yuxuan Liang, Yucheng Liang

**Affiliations:** 1 Center for Clinical Medical Humanities, The Seventh Affiliated Hospital, Sun Yat-sen University, Shenzhen, Guangdong, China; 2 Affiliated Mental Health Center & Hangzhou Seventh People’s Hospital, Zhejiang University School of Medicine, Hangzhou, Zhejiang, China; 3 School of Public Health (Shenzhen), Sun Yat-sen University, Shenzhen, Guangdong, China; 4 Department of Sociology and Social Work, School of Sociology and Anthropology, Sun Yat-sen University, Guangzhou, Guangdong, China; Trinium Woman's Hospital, KOREA, REPUBLIC OF

## Abstract

Alcohol use disorder (AUD) is a growing public health issue which has caused global concern. Previous evidence has identified several genes significantly associated with alcohol-related traits. However, it remains unclear whether these associations are robust across different ethnic groups and whether they may be moderated by some specific social factors. The current study aimed to investigate the associations between candidate SNPs and AUD and to examine whether the associations could be moderated by socioeconomic status and social environment among a cohort of young Chinese males. A cross-sectional survey using convenient sampling was conducted in 2017 in four communities of Guangzhou, China. The current cohort consists of 320 male drinkers aged 18–31 years. Logistic regression was employed to explore the influence of candidate SNPs on AUD. And then, moderation regression model was constructed to investigate the potential moderation effects of multiple social factors measured by attitudes towards alcohol (ATA), personal income level, work-related stress, peer drinking behaviors, and childhood traumas. Of the ten candidate SNPs incorporated in the current study, four (*ALDH2* rs671, *COMT* rs165774, *OPRK1* rs6473797, and *GABRA2* rs279858) were significantly associated with AUD. Moderation analyses revealed that the protective effect of the minor allele of *ALDH2* rs671 was moderated by ATA; the effect of *COMT* rs165774 was moderated by personal income level; and childhood trauma moderated the association between *OPRK1* rs6473797 and AUD. Additionally, *COMT* rs165774 moderated the relationship between work-related stress and AUD risk. This study closely aligned with previous research conducted in Chinese populations and highlighted the importance of considering both genetic and environmental factors in AUD research.

## Introduction

Alcohol use disorder (AUD) is a prevalent social and medical health issue faced with great challenge in early prevention. The detrimental impacts of AUD including multiple chronic diseases and injuries in different dimensions are escalating worldwide [1]. AUD accounts for 5% of disability-adjusted life years [2]. Men with AUD are at twice of the all-cause mortality risk relative to those non-alcohol-dependent [3]. In China, where drinking behavior is special in demographic characteristics and social culture, AUD among males is highly concerned as a pressing health problem [4,5]. The prevalence of AUD in Chinese adult males was estimated to be 10.1% [5]. Many studies suggested that AUD are fairly heritable and have genetic susceptibility [6–8]. It was also reported that about 50% the risk for developing AUD is due to genetics, while the remaining percent may be due to either environmental factors, or gene-environment interactions [9]. As alcohol addiction is affected by genetic disposition combined with environmental risk factors, it is essential to comprehensively examine the behavioral mechanism of AUD with population- and gender-specific study design.

Many certain genes have been explored as candidates for AUD by candidate-gene studies and GWASs. A majority of candidate-gene studies are based on knowledge about the metabolic and neurobiological mechanisms underlying potential genetic risk variants. As a genetic marker related to alcohol metabolizing enzyme, gene Alcohol Dehydrogenase 1B Beta Polypeptide (*ADH1B*) is most commonly associated with AUD. The *ADH1B* rs1229984 minor allele has a protective effect for AUD development, which was found in populations of Asian and American of European and African ancestries [10–13]. The SNP rs671 in gene Aldehyde Dehydrogenase 2 Family Member (*ALDH2*) could lower the rate of metabolizing acetaldehyde, a major metabolite of alcohol, accounting for alcohol flushing especially in East Asians [14]. Thus, the variant of *ALDH2* has been associated with a decreased risk of AUD [15,16]. *ADH* and *ALDH* genes associated with several alcohol-related traits were also found by GWAS in different populations [10,11,17–19].

In addition to alcohol metabolizing enzyme genes, study has also suggested the opioid receptor genes played potential roles in the development of AUD, given that the opioid receptor regulates the pathway that with level of endogenous opioids increasing, the reward system can elicit seeking additional alcohol [20]. The functional variant in gene Opioid Receptor Mu 1 (*OPRM1*) such as rs1799971 may have an effect upon the risk of AUD in different severities [21–23]. Meta-analyses indicated that the *OPRM1* rs1799971 may contribute to the susceptibility of AUD in Asians [24]. A family-based study in European Americans demonstrated that variations in k-opioid receptor gene (*OPRK1*) were associated with AUD [25], which was also found in individual level study in Turkish male population [26]. Besides, dopaminergic genes could be crucial in determining AUD symptoms. Catechol-O-Methyltransferase (*COMT*) is primarily responsible for cortical dopamine inactivation. The effects of *COMT* on AUD has been widely demonstrated, and evidence showed applying *COMT* inhibitor could significantly reduce the AUD symptoms [27,28].

The SNPs involved in alcohol-related traits also interact with individual’s cognition to alcohol drinking behavior. When a low level of response to alcohol predicts future alcohol use disorders in humans, the SNPs (rs279869, rs279858, and rs279837) in *GABRA2*, the γ-aminobutyric acid A (*GABAA*) receptor α2 subunit gene, showed significant associations with subjective effects of alcohol [29].

It is plausible that the candidate genes affect AUD through moderation effect of social factors. However, most previous studies focused on endogenous pathway rather than the external pattern of genetic effect which is more likely to be modifiable. Very few of them are closely related to daily life behaviors and directly examined as moderating mechanism. As published genome-wide data regarding alcohol abuse and dependence are not common in Chinese populations [30], additional work is needed to determine if there is social factor as novel moderator of genetic association to AUD, which remains significant in exploring intervention measures against problematic alcohol-related behaviors.

In this study, we examined the association between the candidate SNPs and AUD by identifying the pattern of genetic effects in a sample of general population in a metropolitan city of South China. Furthermore, we investigated the potential moderation effects of social factors on the associations between SNPs and AUD. The current study focused on male youths to avoid the deviance caused by gender disparities in alcohol-related risks and to help explore more targeted measures of early prevention.

## Methods

### Participants

The participants consisted of 427 unrelated Chinese male youths aged 18–31 years by convenience sampling from general population. The survey was conducted in four sub-districts in Guangzhou, a metropolis at the heart of the Guangdong–Hong Kong–Macau Greater Bay Area in southern China. The recruitment was conducted from March 20^th^ to June 10^th^, 2017. The inclusion criteria for the study participants were as follows: male, age above 18 and below 31 years, living in the city for at least one week, being voluntary to participate in this study and provide saliva sample for genotyping. The exclusion criteria for the study participants were as follows: failing to complete the questionnaire, cannot provide the saliva sample that meet the collection criteria. All participates were recruited from nearby communities of each survey spot with street-intercept method. Prior to participation, they were ensured of fully knowing the details and requirements of the study and signed written informed consent. They were also economically compensated with 100 CNY for completion of face-to-face interview and provision of saliva sample for DNA extraction. The saliva of each participant was collected with special device provided from DNA Genotek Inc. The study was conducted in accordance with the local legislation and institutional requirements. The Ethics Committee of the Department of Sociology and Social Work at Sun Yat-sen University approved the study protocol (Ref. No. 20170206).

## Measures

### Alcohol use disorder (AUD)

We measured AUD using the Alcohol Use Disorders Identification Test (AUDIT), a self-report screening tests developed by the World Health Organization (WHO) [31]. AUDIT is considered as the gold standard in primary care for screening for risk drinking and alcohol addiction. Nowadays it is increasingly used in study for general populations [32–35]. The scale consists of 10 items covering three symptom areas: hazardous use, dependence symptoms, and harmful use. All item scores range from 0 to 4 and are summarized to provide an overall measure. AUD was defined as a binary trait with 8 as lower cutoff point proposed to be adequate for identifying hazardous drinking and alcohol problems [36,37]. The AUDIT questionnaire was utilized to define AUD participant; thus, our following analyses were performed using the binary variable “AUD or not AUD”.

### Attitudes towards alcohol (ATA) and social factors

The major factor of alcohol-related cognition was measured by the fourth version of “Scale for the Measurement of Attitudes Towards Alcohol” [38]. It is a 5-point Likert scale for evaluating the risk profile in relation to alcohol consumption particularly in the age group between adolescence and adulthood. In the fourth version, there are 15 items indicating the predisposition for risky alcohol consumption in three domains: “social ease”, “unease” and “economic aspects”. Respondents were asked about the extent to which they agreed with the statement of each item. The lowest score corresponds to “absolutely disagree” and the highest score corresponds to “absolutely agree”. Sum for the scale indicated respondent’s overall attitude towards alcohol. A higher score represents more positive tendency on alcohol use.

We also examined the effect of socioeconomic and social environmental factors, including personal monthly income, work-related stress, childhood traumas, and peer drinking behavior. The personal monthly income was rated in 10 levels for every 1000 CNY when lower than 10,000, four higher levels for every 5000 CNY from 10,000 to 29,999, until that 30,000 CNY or above denotes the highest single level. Thus, there are 15 levels for the measure of personal income in total. The work-related stress was measured by nine items from two domains referring to “the negative relations with colleagues” and “the failure to achieve job goals” [39]. “The negative relations with colleagues” comprised three items: “conflicts with workmates”, “poor relationship with managers”, and “isolation from workmates”. For “the failure to achieve job goals”, there are six items including “income instability”, “boredom at work”, “stress at work”, “unfair treatment in the workplace”, “the gap between expected and actual returns from work”, and “the failure to get the desired job”. In each item, the respondents were asked to indicate their frequency of being troubled by the issue in the past two years using a 4-point Likert scale ranging from 1 = “never” to 4 = “always”. The sum of each item score was used to measure the work-related stress. We measured the childhood traumas with five stressful events: “parental separation/divorce, parental bereavement, impoverished home environment, parental violent treatment, living involuntarily with a foster family”. The number of events that the respondents experienced in childhood was assessed as a count variable with value ranging from 0 to 5. Also, we investigated peer effects through the question: “How many of your friends engage in alcohol drinking?” (scored from 1 = “none” to 4 = “many”).

### Genotyping and quality control

According to the previous literature, the study incorporated 10 candidate SNPs of four alcohol-related genes, including *COMT* (one intron variant rs165774 and one missense variant rs4680), *OPRK1* (one intron variant rs6473797 and a synonymous variant rs1051660), *GABRA2* (two intron variants rs279845 and rs279871, one missense variant rs279858), *ALDH2* (a missense variant rs671), *OPRM1* (a missense variant rs1799971), and *ADH1B* (a missense variant rs1229984). DNA extraction and genotyping service was provided by Wuhan BGI Medical Laboratory Co., Ltd. The SNP genotyping was performed with MALDI-TOF mass spectrometry, a high-throughput genotyping technology, using the MassARRAY Platform from Agena Bioscience (formerly Sequenom, Inc., San Diego, CA) [40]. Range of 200 bp before and after the target SNP locus was selected to design primers. Locus-specific multiplex polymerase chain reaction (PCR) was then conducted to amplify the sequence. The thermal cycling procedure for PCR amplification comprised 1 cycle in initial denaturation (94°C for 5 min), 45 cycles in denaturation (94°C for 20 sec), annealing (56°C for 30 sec), and extension (72°C for 1 min), 1 cycle in final extension (72°C for 3 min), and a final step in indefinite hold time at 4°C. With the use of MALDI-TOF mass spectrometry, the mass of extended primer is determined to indicate the sequence and automatically translated into a genotype of each reaction by MassARRAY Typer4.0 software (Primers were shown in the Supplementary Table S1).

Quality control was performed according to the standard protocol suggested by Anderson et al. [41]. The genotype distribution of candidate SNPs did not deviate from the Hardy-Weinberg equilibrium. Among the 427 saliva samples, 421 met the quality requirement for genotyping. And six case samples were further removed due to the missing data rate greater than 5%. Thus, the final dataset for analysis included 415 samples with 10 AUD-related SNPs.

### Statistical analyses

To screen for significant SNPs, association analysis was conducted using additive model in logistic regression adjusted by age. Each candidate SNP is coded by the number of minor alleles in genotype (homozygote of major allele = 0, heterozygote = 1, homozygote of minor allele = 2). Bonferroni correction P-value < 0.005 (0.05 divided by 10 as the number of candidate SNPs) was applied to adjust for multiple testing. All corrected P-values in association test were two-sided. The analysis was performed in *PLINK* 1.9 [42].

**Fig 1:**
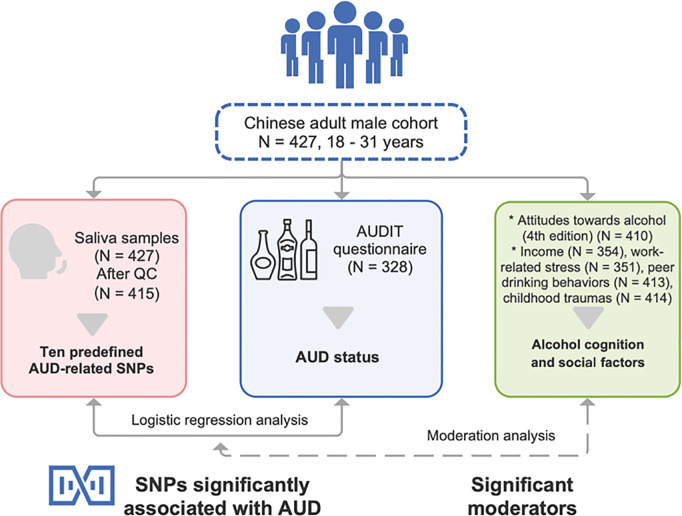
The flow diagram of the current study.

A moderated multiple regression analysis was conducted to investigate the effects of candidate SNPs and other factors on participants’ AUD as target phenotype. For each model, one SNP and one social or genitive factor were entered as independent variable, as well as the interaction term between them. And age was entered as control variable. The regression models were analyzed by *R* 4.2. The flow diagram of the current study was shown in Fig 1.

## Results

### Demographic characteristics

A total of 415 cases were included in the study with an average age of 24.01 ± 3.50 years. The personal income level was reported by 354 participants being employed in the past month, and the average was 5.71 ± 3.04 (Level 5 represents monthly income as 4,000–4,999 CNY). The frequencies of work-related stress were reported by 351 participants who had been in employment in the past two years, with an average of 17.99 ± 5.93. 410 cases reported their ATA with average score of 30.20 ± 7.20. The AUD scale was only applicable to 328 participants who reported to have ever got at least one of following experiences: losing control of drinking too much, being advised to drink less by family members or friends, feeling guilty about drinking, only having a drink very time before breakfast to feel comfortable. There are 150 of them (45.73%) classified as AUD. Details were shown in Table 1.

**Table 1:** Variable Information of Analytic Sample.

Variable	%/ mean±SD	N
age	24.01 ± 3.50	415
income level	5.71 ± 3.04	354
work-related stress	17.99 ± 5.93	351
ATA	30.20 ± 7.20	410
peer drinking behavior	3.27 ± 0.80	413
childhood traumas	0.51 ± 0.84	414
AUD	45.73	328

ATA, attitudes towards alcohol; AUD, alcohol use disorder.

### Significant SNPs associated with AUD

After Bonferroni correction, four SNPs out of ten were found significantly associated with AUD (Table 2). *ALDH2* rs671 showed the highest significance level among all candidates (OR = 0.42, P = 1.462 × 10^−5^). *COMT* rs165774, *GABRA2* rs279858 and *OPRK1* rs6473797 were also still significant in association with AUD status (P = 0.0012, 0.0023, 0.0037; OR = 0.437, 0.630, 0.611). The other six SNPs (*GABRA2* rs279871, *GABRA2* rs279845, *OPRK1* rs1051660, *COMT* rs4680, *OPRM1* rs1799971, *ADH1B* rs1229984) were not significant for the corrected P-value threshold (P > 0.005).

**Table 2:** List of Candidate SNPs and Effects on AUD.

rsID	Gene	Chr.	Position	A1	A2	FRQ(A1/A2)	OR	SE	P-value
rs671	*ALDH2*	12	111803962	A	G	186/395	0.420	0.200	**1.462 × 10** ^**−5**^ *
rs165774	*COMT*	22	19965038	A	G	97/411	0.437	0.256	**1.200 × 10** ^**−3**^ *
rs4680	*COMT*	22	19963748	A	G	197/385	0.742	0.168	7.542 × 10^−2^
rs6473797	*OPRK1*	8	53240422	C	T	247/382	0.611	0.170	**3.716 × 10** ^**−3**^ *
rs1051660	*OPRK1*	8	53251002	A	C	114/409	0.618	0.229	3.537 × 10^−2^
rs279858	*GABRA2*	4	46312576	T	C	289/339	0.630	0.152	**2.331 × 10** ^**−3**^ *
rs279871	*GABRA2*	4	46303716	T	C	274/322	0.653	0.152	5.055 × 10^−3^
rs279845	*GABRA2*	4	46327706	T	A	265/355	0.653	0.153	5.436 × 10^−3^
rs1799971	*OPRM1*	6	154039662	G	A	247/374	0.736	0.170	7.209 × 10^−2^
rs1229984	*ADH1B*	4	99318162	C	T	193/394	0.949	0.169	7.582 × 10^−1^

A1, minor allele; A2, major allele; FRQ(A1/A2), frequencies of A1 and A2.

* significant P-value after Bonferroni correction (p < 0.05/10).

### Moderation effect of social factors on genetic associations of AUD

The results of moderated multiple regression analyses were shown in Table 3. We identified significant moderation effects of ATA, socioeconomic factors, and childhood traumas experience on SNPs’ effects to AUD.

**Table 3:** Interactions between SNPs and social factors on AUD.

Variable	β	SE	z	P-value
*ALDH2* rs671	−3.272	1.203	−2.72	0.007**
ATA	0.015	0.022	0.69	0.488
ATA ×*ALDH2* rs671	0.081	0.037	2.16	0.031*
age	−0.021	0.034	−0.63	0.528
*COMT* rs165774	−2.034	0.675	−3.01	0.003**
income level	0.040	0.050	0.81	0.417
income level × *COMT* rs165774	0.220	0.095	2.31	0.021*
age	−0.089	0.042	−2.09	0.037*
*COMT* rs165774	1.479	0.919	1.61	0.107
work-related stress	0.071	0.025	2.77	0.006**
work-related stress × *COMT* rs165774	−0.122	0.048	−2.54	0.011*
age	−0.038	0.037	−1.03	0.301
*OPRK1* rs6473797	−0.756	0.228	−3.32	0.001**
childhood traumas	−0.589	0.222	−2.65	0.008**
childhood traumas × *OPRK1* rs6473797	0.614	0.227	2.71	0.007**
age	−0.005	0.033	−0.17	0.868

‘AUD, alcohol use disorder; ATA, attitudes towards alcohol.

*p < 0.05, ** p < 0.01, *** p < 0. 001.

**Fig 2:**
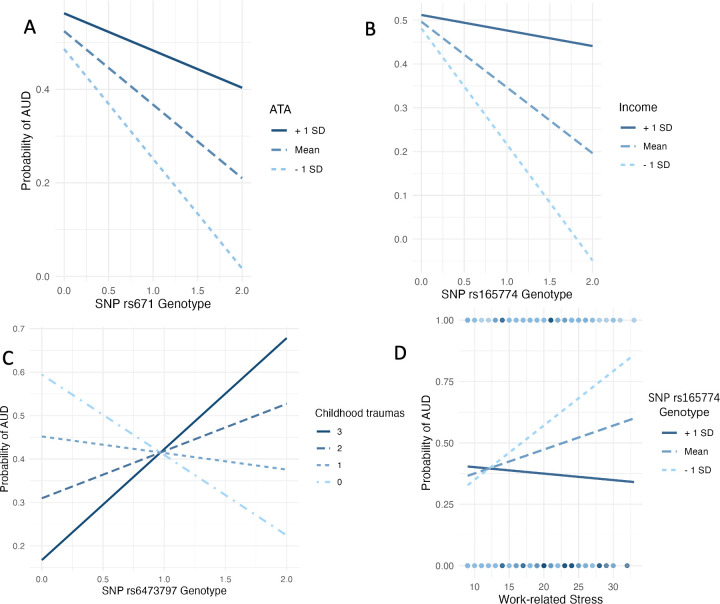
The Moderation Effects on the Association between SNPs and AUD. (A) The moderation effect of attitudes towards alcohol use (ATA) on the association between SNP *ALDH2* rs671 and AUD; (B) The moderation effect of income level on the association between *COMT* rs165774 and AUD; (C) The moderation effect of childhood traumas on the association between *OPRK1* rs6473797 and AUD; (D) The moderation effect of *COMT* rs165774 on the relationship between work-related stress and AUD.

For the model with interaction term between *ALDH2* rs671 and ATA, the main effect of rs671 was significant (β = −3.27, SE = 1.20, P < 0.01), whereas the main effect of the overall attitude score was not significant (β = 0.02, SE = 0.02, P = 0.49 > 0.05). It was also revealed that the moderation effect was significant (β = 0.08, SE = 0.04, P < 0.05) (Table 3, Fig 2). These results indicated that the association between *ALDH2* rs671 and AUD was moderated by ATA. Specifically, the ATA would weaken the protective effect of minor allele in *ALDH2* rs671 on AUD.

For the model testing interaction of *COMT* rs165774 and income level, the main effect of *COMT* rs165774 was significant (β = −2.03, SE = 0.67, P < 0.01). The main effect of the income level was not significant (β = 0.04, SE = 0.05, P = 0.42 > 0.05), but the moderation effect was significant (β = 0.22, SE = 0.10, P < 0.05) (Table 3, Fig 2). According to these results, we can find that personal income would also weaken the protective effect of *COMT* rs165774. It is also indicated that the carriers of the minor allele in *COMT* rs165774 who had relatively high income levels were less likely to develop AUD.

The test of moderated regression also revealed that the interaction effect between *COMT* rs165774 and work-related stress was significant and positive. The main genetic effect was found insignificant, while the main social effect was found significant. The results altogether indicated that the *COMT* rs165774 probably relieved the risk of AUD affected by work-related stress.

Finally, we examined the potential moderating role of childhood trauma on the genetic association between *OPRK1* rs6473797 and AUD. Our results suggested that higher levels of childhood trauma may attenuate the protective effect of the *OPRK1* rs6473797 effect allele on AUD risk (β = 0.614, SE = 0.227, P < 0.01).

## Discussion

The current study examined the associations between candidate SNPs and alcohol use disorder (AUD) in a Chinese male cohort. Our findings closely aligned with previous research conducted in Chinese populations and further explored the moderating role of social factors. The results showed that the candidate SNPs, *ALDH2* rs671 and *COMT* rs165774, were associated with AUD in different patterns of moderation effect. The ATA has a moderation effect on the relationship between *ALDH2* rs671 and AUD, which means that more extent of positive attitude to alcohol drinking may contribute to less benefits of genetic inheritance for avoidance the alcohol abuse. The personal income moderated the association between *COMT* rs165774 and AUD by hindering the protective effect of minor allele of *COMT* rs165774. Childhood trauma attenuated the protective effect of the minor allele of *OPRK1* rs6473797 on AUD, suggesting that individuals with greater exposure to childhood trauma may be more vulnerable to developing AUD. Interestingly, *COMT* rs165774 also moderated the effects of work-related stress on AUD.

For the *ALDH2* rs671, the current results are consistent with the evidence from GWAS study demonstrating its protective effect on AUD in Chinese males [18], and several candidate gene studies [43,44]. A recent large-scale study (168,050 participants) confirmed the association between *ALDH2* rs671 and alcohol consumption, which is highly relevant to AUD status, in Chinese population, and found it relevant to multiple comorbidities includes liver cirrhosis, stroke and gout [45]. The current study further demonstrated that the attitude towards alcohol use could influence the effects of *ALDH2* rs671 on AUD. One potential reason cannot be neglected is that some genes related to AUD could also affect the attitudes towards alcohol. The finding implies that more gene expression studies are required to broaden the exploration in associations among genetics, epigenetics, and nutrition which may simultaneously affect AUD and its behavioral factors [46]. As studies in Asian population reported that facial flushing is a direct mechanism by which the frequency of alcohol consumption is reduced [15], early intervention should focus more on the health risk education to restrain the irrational ideology to the social benefits of alcohol behavior.

For the *COMT* rs165774, its positive effect against the AUD was found to be hindered by personal income while alleviating the risky effect of work-related stress on AUD. Such complexity in different patterns of moderation effect elaborated the partial effect of *COMT* activity on alcoholism suggested by prior study in other Asian population [47]. It may also reveal the potential reason why *COMT* polymorphism was not associated with alcohol dependence in Korean population [48]. The double moderation implies that income may play a more negative role than work-related stress in development of AUD when considering the genetic influence in it. This is consistent with the goal of social equality from the perspective of behavioral health.

Compared to previous studies mainly concerning the physiological response as the phenotype of alcohol related traits, this study not only largely supports their findings in a different Asian group with specific age level and gender characteristics, but also extends the understanding of genetic mechanisms of alcohol abuse and dependence to psychological and social domain.

There are limitations in this study. Firstly, the current study utilized a limited and convenience-based sample, which could introduce potential selection bias and compromise the generalizability of findings. Self-selection recruitment often overrepresents motivated or accessible subgroups while underrepresenting marginalized populations. This would limit their applicability to broader populations, particularly for policy contexts where equitable representation is critical. Future studies with larger, more diverse populations and rigorous sampling strategies are needed to validate and expand upon these results. Secondly, as the number of candidate SNP is not in a large scale like GWASs, the study cannot reveal novel genetic locus and more pattern of association between different domains of factors and alcohol abuse. Further studies on larger sample size and more candidate genes are needed to substantially provide more power of statistics in association to the alcohol-related traits with more diverse patterns in both moderation and mediation. Thirdly, while family and social support networks have been demonstrated to influence AUD individuals [49], our study did not assess these factors, which might limit our understanding of how social context interacts with the symptoms. In the current study, we only measured “peer drinking behavior” to investigate the effects of friends’ drinking behavior but did not find significant moderation effect on the associations between candidate SNPs and AUD. Besides, the measurement is rather coarse, and given existing evidence that family drinking behavior can also influence individuals’ AUD status, future studies may benefit from examining both peer and family influences using more fine-grained approaches. Future research would benefit from incorporating standardized measures of social support. Finally, the cross-sectional design of this study inherently restricts our ability to establish any causal relationships. While our results revealed interesting associations that align with existing evidence, they should be interpreted as correlational rather than causative. Future longitudinal studies with randomized sampling approaches would be valuable for verifying these preliminary observations and exploring potential causal mechanisms. In particular, longitudinal incidence surveys are crucial for tracking the onset, progression, and temporal patterns of AUD, which can help distinguish between predictors and outcomes, promote better identification of risk trajectories and facilitate the development of targeted, time-sensitive interventions.

## Conclusion

AUD represents a complex phenotype shaped by dynamic gene-environment interactions, with significant variations across populations and genders. Focusing on a Chinese male youth cohort from metropolitan city, this study identified significant associations of exon variant in *ALDH2* and intron variants in *COMT* and *OPRK1* with AUD, aligning with previous findings in other East Asian populations. Furthermore, the current study also demonstrated the moderation effects of different social factors on these genetic effects, offering actionable insights for personalized risk mitigation strategies. To conclude, the findings reinforce the role of key genetic factors in AUD and provide insights that could aid in the primary prevention of AUD among high-risk groups.

## Supporting information

S1 Table PCR primers for SNP genotyping on the MassARRAY platform. (DOCX)
