# Supplementary material for: The interaction between candidate SNPs and social factors significantly influences the prevalence of alcohol use disorder in Chinese young male adults
Source: PLoS One. 2025 Aug 26;20(8):e0330822. doi: 10.1371/journal.pone.0330822 (PMC12380350; doi:10.1371/journal.pone.0330822)
Supplement: S1 Table — (DOCX) [file pone.0330822.s001.docx]

**S1 Table. PCR primers for SNP genotyping on the MassARRAY platform.**

| SNP | PCR primers | Extension primer |
| --- | --- | --- |
| rs671 | F: ACGTTGGATGGAGTGGCCGGGAGTTGGG | AGTACGGGCTGCAGGCATACACT |
|  | R: ACGTTGGATGAAGCCCCAACAGGCCCTG |  |
| rs165774 | F: ACGTTGGATGGCTCGCTCACCTGCAGCT | AGAAACTGGACACTGCTGTTAGCAGCC |
|  | R: ACGTTGGATGTCCAGCGGCCCTACCTAG |  |
| rs4680 | F: ACGTTGGATGCCCGACTGTGCCGCCATCA | GCGGATGGTGGATTTCGCTGGC |
|  | R: ACGTTGGATGCCAGGTCTGACAACGGGTCAG |  |
| rs6473797 | F: ACGTTGGATGGCACCTGAACAAATGCTGT | GTGAAAACACAAGTGTGATCAAATGCCA |
|  | R: ACGTTGGATGGACTCCAAGGGCAGCTCTA |  |
| rs1051660 | F: ACGTTGGATGCAGGCGCTGCTGTTGGG | CGGGGCGCAGGTAGGGCC |
|  | R: ACGTTGGATGCTGCTGCAATCGCCCCACC |  |
| rs279858 | F: ACGTTGGATGTGAATTCGAAGCAACTTATTTG | GCATTGTCATATTATGAGCTACTGATTT |
|  | R: ACGTTGGATGCAATTTAATGGCTAGCAAAATCTG |  |
| rs279871 | F: ACGTTGGATGTAGGAAATAGAAGGGATCAG | GGTAGAACAAAAACTGATTTTTTAAAAAA |
|  | R: ACGTTGGATGGTGATATAAATTTCCTGACATG |  |
| rs279845 | F: ACGTTGGATGCCTTGATATTAACTACTGAAC | TTAATATCCCAGTAGCTTCTGGAG |
|  | R: ACGTTGGATGAAACACAGAGTGCATTATC |  |
| rs1799971 | F: ACGTTGGATGAGCACCCAGCCCCGGTT | CTGGGTCAACTTGTCCCACTTAGATGGCA |
|  | R: ACGTTGGATGCCCAGGTCGGTGCGGTTC |  |
| rs1229984 | F: ACGTTGGATGTCACAGGAAGGGGGGTCA | TGCCACTAACCACGTGGTCATCTGTG |
|  | R: ACGTTGGATGCTCTTTATTCTGTAGATGGTGGC |  |

F, forward; R, reverse
